# The Unfulfilled Promise of Inhaled Therapy in Ventilator-Associated Infections: Where Do We Go from Here?

**DOI:** 10.1089/jamp.2021.0023

**Published:** 2022-02-14

**Authors:** Lucy B. Palmer, Gerald C. Smaldone

**Affiliations:** Pulmonary, Critical Care and Sleep Division, Department of Medicine, Stony Brook University, Stony Brook, New York, USA.

**Keywords:** aerosolized antibiotics, bacterial resistance, ventilator-associated pneumonia

## Abstract

Respiratory infection is common in intubated/tracheotomized patients and systemic antibiotic therapy is often unrewarding. In 1967, the difficulty in treating Gram-negative respiratory infections led to the use of inhaled gentamicin, targeting therapy directly to the lungs. Fifty-three years later, the effects of topical therapy in the intubated patient remain undefined. Clinical failures with intravenous antibiotics persist and instrumented patients are now infected by many more multidrug-resistant Gram-negative species as well as methicillin-resistant *Staphylococcus aureus*. Multiple systematic reviews and meta-analyses suggest that there may be a role for inhaled delivery but “more research is needed.” Yet there is still no Food and Drug Administration (FDA) approved inhaled antibiotic for the treatment of ventilator-associated infection, the hallmark of which is the foreign body in the upper airway. Current pulmonary and infectious disease guidelines suggest using aerosols only in the setting of Gram-negative infections that are resistant to all systemic antibiotics or not to use them at all. Recently two seemingly well-designed large randomized placebo-controlled Phase 2 and Phase 3 clinical trials of adjunctive inhaled therapy for the treatment of ventilator-associated pneumonia failed to show more rapid resolution of pneumonia symptoms or effect on mortality. Despite evolving technology of delivery devices and more detailed understanding of the factors affecting delivery, treatment effects were no better than placebo. What is wrong with our approach to ventilator- associated infection? Is there a message from the large meta-analyses and these two large recent multisite trials? This review will suggest why current therapies are unpredictable and have not fulfilled the promise of better outcomes. Data suggest that future studies of inhaled therapy, in the milieu of worsening bacterial resistance, require new approaches with completely different indications and endpoints to determine whether inhaled therapy indeed has an important role in the treatment of ventilated patients.

## Introduction

Recent systematic reviews of inhaled antibiotics as therapy for ventilator-associated infections (Table 1) demonstrate substantial heterogeneity in terms of indications, endpoints, devices, antimicrobials, and doses of antibiotics.^(1–4)^ Despite this heterogeneity, substantial interest remains in this form of therapy culminating in two large randomized trials of adjunctive inhaled antibiotics.^(5,6)^ Both failed to show more rapid resolution of symptoms or a mortality effect.

**Table 1.** Summary of Meta-Analyses of Clinical Trials of Inhaled Therapy for Ventilator-Associated Pneumonia

Imprecise definitions of the infection being treated^(1)^
No consistency in dosing^(1,2,4)^
No control of drug delivery devices^(1,4)^
Imprecise outcomes such as clinical cure^(2–4)^
Unrealistic outcomes such as attributable mortality^(2)^

Commentaries and editorials discussing these particular trials mentioned possible weaknesses with devices and drug delivery, the choice of the experimental population and endpoints, and they suggested a re-evaluation of the design of future trials.^(7–11)^ The core observations listed in Table 1 suggest that problems in the field are fundamental and indicate a certain irrationality in design and outcomes. Repeated similar studies may not succeed. In this review, we call for a re-exploration of how to treat ventilator-associated infections.

Clinical trials should include new treatment algorithms and new endpoints. We address the unique problems of treating respiratory infection in the ventilated patient specifically revisiting airway pathophysiology and the current definitions of respiratory infection in the presence of the endotracheal tube. Contrary to decreasing mortality, or clinical cure of established ventilator-associated pneumonia (VAP), we argue that goals for future investigations should include preventing VAP thus reducing systemic antibiotic use and interrupting the continuing emergence of increasingly resistant pathogens in the ICU.

## What Is Respiratory Infection in the Critically Ill Ventilated Patient?

Ventilator-associated infections are fundamentally different than pneumonia in a spontaneously breathing patient. The endotracheal tube causes localized persistent inflammation that favors localized infection and interrupts multiple host defenses such as mucociliary clearance and cough. Like a splinter in the skin, this localized inflammation/infection will not resolve until the foreign body is removed. Furthermore, all the signs and symptoms associated with pneumonia in the spontaneously breathing patient become nonspecific in the ventilated patient.

Fever, radiographic infiltrates, and increased secretions are common and may not be caused by lung infection. It is not surprising that there is no gold standard for defining ventilator-associated infections. For example, even the seemingly objective measure of quantification of bacterial colony forming units from bronchial lavage fluid (BAL) is highly sensitive and specific only if patients have no prior antibiotic exposure, have been on a ventilator <21 days, and if the BAL followed a standardized technique.^(12–15)^ These methodological weaknesses were acknowledged in the most recent American Thoracic Society/Infectious Disease Society of America (ATS/IDSA) Guideline from 2016, which did not recommend BAL for the diagnosis of pneumonia.^(16)^

**FIG. 1.**
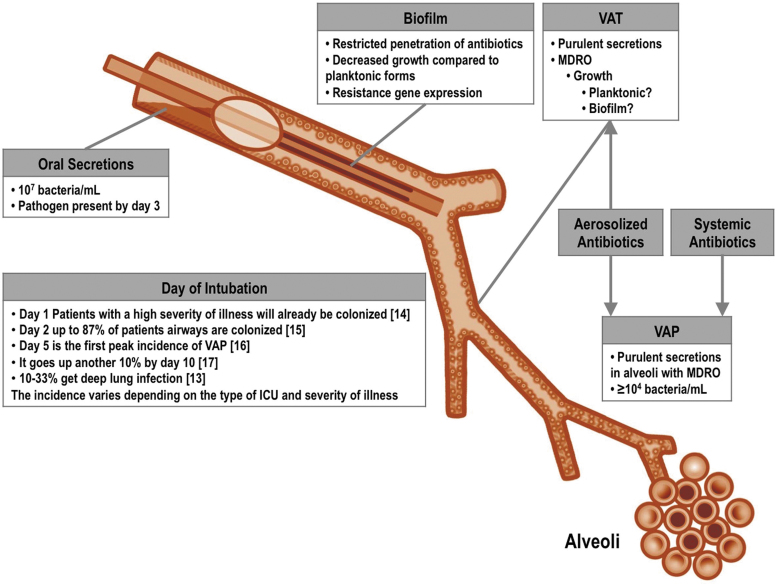
Pathophysiology of respiratory infection in the intubated patient. MDR pathogens colonize the oropharynx of critically ill patients before or soon after intubation. After colonization of the oropharynx occurs, oral secretions then pool near the cuff and organisms enter the proximal airway directly from microaspiration. Shortly after the placement of the endotracheal tube, there is localized injury to the mucosa near the cuff, and mucociliary clearance is dramatically impaired. These processes remain as long as the patient is intubated. In addition, biofilm may form within the tube and the airways act as a constant reservoir of organisms that may be displaced into the lung with suctioning and saline instillation. Bacteria in this reservoir may not be treated adequately with systemic antibiotics. (Modified from Aerosolized antibiotics for ventilator-associated infections. Chapter 10.4. In: Dhand R, editor: Textbook of aerosol medicine. Knoxville TN: International Society of Aerosols in Medicine; 2015. p.1–28.). MDR, multiple drug resistant.

Figure 1 describes proposed sequential steps leading to deep lung infection after intubation.^(16–20)^ After a few days of mechanical ventilation, there is pathogenic colonization of the mouth, aspiration into the airway, followed by bacterial growth that may lead to tracheobronchitis and deep lung infection. Community-acquired pneumonia involves colonization, microaspiration, and/or inhalation but does not involve a foreign body and in most cases cough is intact. The path to ventilator-associated infection differs in multiple significant ways: including the type of organisms that initiate the process, the persistent airway inflammation and epithelial injury from the endotracheal tube, the inability to clear secretions effectively due to loss of cough, impairment of mucociliary clearance, and frequent micro- and macroaspiration.

**FIG. 2.**
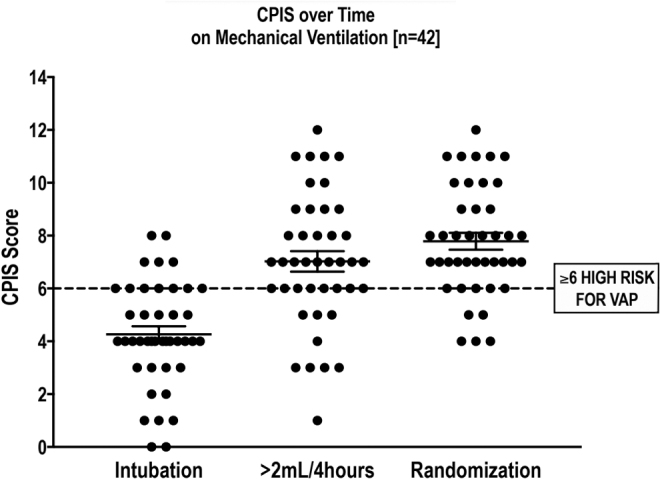
CPIS progression in the intubated patient. This figure shows CPIS in a group of newly intubated patients (from a clinical trial over time before any inhaled therapy. As shown, there were highly significant increases over time.^(21)^ CPIS, clinical pulmonary infection score.

Figure 2 shows serial clinical data from newly intubated patients qualitatively consistent with the model depicted in Figure 1.^(21,22)^ As inflammation in the airways progresses, clinical signs emerge such as purulent secretions, low-grade fever, and increasing white blood cell count that all contribute to a clinical picture described by the clinical pulmonary infection score (CPIS).^(22,23)^ CPIS rapidly rises over time consistent with the chronological progression of infection emanating from the foreign body. Clinicians react to the increasing CPIS (or its components) by starting systemic antibiotics often without a definitive diagnosis. In this context, what treatment plan will be effective? And what are the appropriate outcomes to measure?

## What Do the Failures of Systemic Therapy Tell Us?

In a recent systematic review of 27 randomized controlled trials (RCTs) for the treatment of hospital-acquired pneumonia/VAP from 1994 to 2016 by Weiss et al. with systemic therapy, the clinical cure rate was on average 54% with a range of 23%–77%.^(24)^ Not only is this metric disappointing, but equally disturbing was also the lack of consistency of the definition of “clinical cure.” Definitions varied including “resolution of signs and symptoms” or “improving clinical signs and symptoms” with no additional antibiotic added during the study period, or in some investigations, there was no delineation of what comprised a clinical cure. Assessment of efficacy is difficult to study if investigators are not measuring the same endpoints.

Despite the poor past record, Weiss et al. indicate that the same failings can be found in contemporary studies actively enrolling patients (Clinical Trials.gov).^(24)^ These have fundamental design problems that may preclude clinical success. For example, many treatment trials use mortality as a primary or secondary endpoint. The recent Phases 2 and 3 trials (IASIS, INHALE), which tested new specially formulated inhaled aerosol formulations, included mortality as a primary endpoint or as part of a hierarchal endpoint.^(5,6)^

Mortality is a discrete unarguable endpoint, but it is unlikely to be an achievable endpoint for the treatment of respiratory infection in the critically ill. VAP leads to prolonged intubation and a general increase in resistant organisms but not usually to high attributable rates of death.^(24,25)^ This failed strategy calls for re-evaluation of why both systemic and adjunctive inhaled therapies have failed to consistently improve outcomes.

## Physiological and Microbiological Effects of Inhaled Versus Systemic Therapy

Our current approach to therapy has intrinsic weaknesses. This is well demonstrated by the rapid emergence of resistance as each new antibiotic goes on the market. Increasingly potent systemic antimicrobials soon lose their efficacy as bacteria rapidly mutate and overcome the unique molecular mechanisms that make the antibiotic more active. This is substantiated by the fact that clinical cure rates have not changed and remain ∼50%. In fact, FDA approval for new systemic antibiotics is based on noninferiority to older antimicrobials with a similar spectrum of bactericidal activity, suggesting that detecting a superior clinical effect is unlikely.

Pre-clinical pharmacokinetic and pharmacodynamic trials for pneumonia using both systemic and inhaled antibiotics are further complicated by the methods used and accepted by the FDA to determine antibiotic concentrations in the lung. Adequate drug concentrations are evaluated by the minimum inhibitory concentration (MIC) or area under the curve for the antibiotic and the pathogen under investigation. For pneumonia, drug exposure in the epithelial lining fluid (ELF) is thought by many to be the proper compartment to assess efficacy of drug delivery whether it be systemic or inhaled delivery. For example, the two recently approved systemic antibiotics for ventilator-associated pneumonia, ceftolazane/tazobactam and ceftazidime/avibactam, reported ELF concentrations in support of successful intravenous delivery.^(26,27)^

Recent trials of inhaled therapy have used ELF concentrations to demonstrate adequate drug delivery as well.^(28,29)^ However, there are now multiple publications suggesting that ELF concentrations are likely to be inaccurate and result in an overestimation of true parenchymal exposure for inhaled therapy.^(30–32)^ Furthermore, there are no human studies indicating that ELF concentrations correlate with clinical outcomes.^(31–35)^ Finally, and perhaps most importantly, concentrations in ELF (an alveolar parameter) do not inform us about a separate compartment of infection, the instrumented airway. Here amid viscous and purulent secretions, drug concentrations may need to exceed 10–25 times the MIC of the pathogen to be bactericidal.^(36)^

In the airway, antibiotics may be either inactivated or exhibit reduced effectiveness, secondary to binding to mucin or other airway proteins or to poor penetration into biofilm.^(37,38)^ In addition, MICs are a moving target for antimicrobial susceptibility over time. The longer a patient is in the ICU receiving systemic antibiotics for any infection, the more difficult lung infections will be to treat. This clinical effect is secondary to alterations in gut and lung microbiomes with increasing antibiotic resistance.^(39–41)^ The presence of these increasingly resistant pathogens led to the trials of adjunctive therapy (inhaled plus systemic). In theory, the combination of both systemic therapy and inhaled therapy should result in high concentrations proximally and distally. Why did this strategy fail?

## Inhaled Delivery of Antibiotics

### The healthy versus the infected lung

In experimental animal models of inhaled and intravenous drug delivery, significant differences are found in models that examine healthy lung versus experimental models of pneumonia. Dhanini et al. studied the pharmacokinetics of inhaled and intravenous tobramycin in a healthy lung ovine model of delivery.^(49)^ In mechanically ventilated healthy sheep, concentrations of tobramycin were measured in ELF and in the interstitial space fluid after inhaled and intravenous antibiotic administration. This model found higher concentrations of inhaled antibiotics in ELF and interstitial fluid than that achieved by intravenous therapy in these noninfected animals.

This observation did not carry over to infected animals. Two experimental models of inhaled delivery in porcine VAP demonstrated that concentrations of antibiotic were higher in animals with less severe pneumonia.^(50,51)^ Goldstein et al. demonstrated that in anesthetized ventilated piglets, tissue concentrations of nebulized amikacin were 3–30-fold greater than those achieved with intravenous therapy.^(50)^ However, in areas of severe lower lobe pneumonia, deposition of amikacin was decreased. Ferrari et. al demonstrated similar results in ventilated piglets.^(51)^ Subjects with the most severe pneumonia had decreased distal deposition of inhaled ceftazidime.

Most recently, Li Bassi et al. describe an elegant porcine model of severe pneumonia.^(52)^ Inhaled amikacin and fosphomycin were compared with IV meropenem alone and combined inhaled and IV therapy. The primary outcome was lung tissue bacterial concentration. Secondary outcomes were tracheal secretions *Pseudomonas aeruginosa* concentration, clinical variables, lung histology, and development of meropenem resistance. Inhaled therapy resulted in more effective bacterial eradication in the tracheal secretions but had negligible effect in lung tissue. Intravenous meropenem was essential for bactericidal activity in the lung parenchyma. Resistance to meropenem increased only in the IV meropenem alone group versus amikacin and fosfomycin + meropenem (*p* = 0.004).

The data are all consistent with our concerns about delivery in well-established infection and may at least partially explain why inhaled therapy lacked robust effects in many trials. It also confirms earlier findings that inhaled therapy protects against resistance caused by systemic antibiotics.

### Review of devices and outcomes in recent trials

Effective inhaled therapy requires use of a delivery device that is well characterized in terms of its particle size, deposition site, the effect of humidity on its function and the concentrations achieved at the site of infection. It also must be robust and insensitive to ventilator settings, delivering the same reproducible amount with each treatment. Non-nebulizer issues include breath actuation, location of the device in the circuitry, and drug formulation.

This review will not detail the available devices, which are well described elsewhere.^(9,53–56)^ However, the application of different devices in recent trials, and effects on clinical and microbiological responses are shown in Table 2.^(21,22,42–44,46–48,57–59)^ Even though the type of device used in a trial may directly affect drug efficacy, most published studies using inhaled therapy in the ICU neither describe the method of aerosolization nor the concentration of drug achieved in the lung or secretions. These studies do not meet the basic criteria for acceptable drug delivery.

**Table 2.** Delivery Device and Microbiological and Clinical Response to Inhaled Antibiotics in the ICU 2008–2019

Authors	Year	Setting	Design	Indication	Drug and Method of Aerosolization: No. of Patients on IA or IV or Placebo	Organisms in Patients	Number of Patients with Eradication of Causative Organism	Number of Patients with Newly Resistant Organisms	Clinical Response	Adverse Events
Palmer et al.^(21)^	2008	ICU, United States	Randomized double-blind placebo controlled	VAT ≥2 mL sputum/4 hours and organism on Gram stain	Vancomycin and/or gentamycin jet nebulizer; placebo-24; 19/24 (79%) also on IV IA-19; 17/19 (89%) also received IV	Multiple species of Gram-negative and Gram-positive organisms	IA 12/12 (100%) isolates at day 14 Placebo; 5/19 (26%) isolates at day 14	Placebo 8/24 (33%), IA 0/19 (0%)	IA vs. placebo Resolution of VAP (adjusted OR, −0.29; 95% CI, 0.13–0.66, *p-* = 0.006). Reduced use of systemic antibiotic *p* = 0.042 Increased weaning *p* = 0.046	No bronchial constriction
Kofteridis et al.^(42)^	2010	ICU, Greece	Retrospective review, matched case control	VAP Clinical diagnosis + endotracheal secretions or BAL	Colistin; Aerosolization—not described IV and aerosolized colistin—43 IV colistin—43	*Acinetobacter*-66 *Klebsiella-12* *Pseudomonas*-8 All susceptible to colistin	IV = 17/34 (50%); IV+IA, 19/42 (45%) *p* = 0.679	No resistance in IA group; Resistance in IV group not described	IA+IV vs. IV Clinical cure *p* = 0.679 Mortality *p* = 0.289	Renal impairment no different in either group No neurotoxicity in either group
Korbila et al.^(43)^	2010	ICU, Greece	Retrospective review, matched case control	VAP Clinical diagnosis and quantitative cultures of respiratory specimens	Colistin Aerosolized through Siemens Servo ventilator, aerosolized colistin+IV-78 IV colistin-43	MDR Gram-negative organisms; *A. baumanii, Pseudomonas aeruginosa, Klebsiella spp.;* All colistin susceptible	Not described	Not described	Cure IV + IA 62/78 (79%) vs. IV = 26/43 (60%) *p* = 0.025 ICU mortality 28/78 (36%) vs. 17/43 (40%) *p* = 0.92	No bronchial constriction
Rattanaumpawan et al.^(44)^	2010	ICU, Thailand	Open label RCT	VAP Clinical diagnosis plus Gram negative in secretions	Colistin Aerosolization—not described IA+IV-51 Placebo+IV—49	Colistin susceptible *P. aeruginosa* and *A. baumanii*	IA+IV 31/51 (61%); Placebo + IV 19/49 (39%), *p* = 0.03	Not described	IA+IV 26/51 (51%); Placebo + IV 25/49 (51%), *p* = 0.84 IA group, shorter days of IV antibiotic	No difference in renal impairment or bronchial constriction
Lu et al.^(46)^	2011	ICU France	Randomized trial comparing IA with IV antibiotics	VAP Clinical diagnosis + BAL or mini-BAL	Vibrating plate nebulizer Nebulized amikacin and ceftazidime *n* = 20 amikacin and ceftazidime IV *n* = 20	*P. aeruginosa* susceptible to drugs	IA - 16/16 (100%) on day 5; IV-7/15 (47%) on day 5	IA day 9, 0/12 (0%) IV day 9, 5/11 (45%)	IA 14/20 (70%) IV 11/20 (55%) *p* = NS	IA-Hypoxemia-3/20 (15%) Expiratory filter occluded-3/20 (15%) 1/20 (5%) cardiac arrest
Arnold et al.^(47)^	2012	MICU SICU United States	Retrospective chart review with cohort study	VAP Clinical diagnosis + BAL	Colistin Vibrating plate Inhaled antibiotics Colistin *n* = 9, tobramycin *n* = 10. All patients also on IV IV only *n* = 74	*P. aeruginosa* and *Acinetobacter baumanii* susceptible to drug administered	Not described	Not described	Increased survival by Kaplan–Meier for IA+IV *p* = 0.030	IA+IV 1 episode of bronchial constriction; 1 episode of renal impairment
Lu et al.^(48)^	2012	ICU, France	Prospective observational comparator	VAP Clinical dx + BAL or blind mini-BAL	Colistimethate; Vibrating plate nebulizer; Three arms; (1) Cohort group with organisms susceptible to β-lactams Tx = IV only *n* = 122 (2) Group with organisms resistant to β-lactams Tx = IV plus aerosol *n* = 15; (3) Group with organisms resistant to β-lactams Tx = aerosol alone *n* = 28	*P. aeruginosa* and *Acinetobacter spp.* β-lactam susceptible *P. aeruginosa* and *A. baumanii*-susceptible to colistin resistant to β-lactams	Not described	Reported for patients with recurrent infection IA 4/16 (25%) converted from β-lactam resistant to susceptible after inhaled therapy IV 24/32 = 75% of isolates developed new resistance 6/32 became resistant to all β-lactams	Clinical cure; β-lactam sensitive 81/122 (66%); β-lactam resistant 29/43 (67%) *p* = NS; Mortality; β-lactam sensitive 28/122 (7%); β-lactam resistant on IA 7/43 (16%) *p* = 0.357	No renal toxicity observed Creatinine similar in both groups
Niederman et al.^(57)^	2012	Multisite Phase 2 trial in United States Spain and France	VAP-clinical diagnosis Proprietary amikacin BAY41-6551 vibrating mesh nebulizer 67 patients divided into three groups	VAP Clinical diagnosis + BAL or tracheal aspirates	All received intravenous antibiotics according to ATS guidelines 2005. Inhaled amikacin^†^ at 400 mg Q 12H and 400 mg Q 24H Placebo—normal saline Q 12H Drug and saline were aerosolized with the proprietary pulmonary drug delivery system	Gram-negative organisms Predominant species—*Pseudomonas*	Inhaled amikacin^†^ 22/33 (68.8%) Placebo 10/16 (62.5%)	Not described	Inhaled amikacin^†^ Q 12, 93.8% (15/16), Inhaled amikacin^†^ Q 24H 75.0% (12/16), and Placebo 87.5% (14/16) (*p* = 0.467) mean number of antibiotics per patient per day Inhaled amikacin^†^ Q12 0.9/day in the q12h, Inhaled amikacin^†^ q24h 1.3/day in the q24h, and Placebo 1.9/day in the placebo groups, *p* = 0.02 between groups	
Doshi et al.^(58)^	2013	Medical Surgical ICUs United States	Retrospective multi-center cohort study	VAP diagnosed by BAL or endotracheal secretions	Colistin Aerosolized with jet nebulizers or vibrating mesh nebulizer; IV only = 51 patients IV plus IA colistin = 44	IV patients: *Acinetobacter* 25/51 (49%) *Pseudomonas* 35/51 (69%) ESBL 9/51 (18%) IV+IA *Acinetobacter* 36/44 (82%) *Pseudomonas* 18/44 (41%) ESBL 2/44 (5%) All organisms susceptible to colistin	IV 27/51 (53%) IV+IA 18/44 (41%) *p* = 0.805	Not described	In patients diagnosed with BAL IV-9/32 (31.3%) IV + IA 19/35 (57%) *p* = 0.033	Not reported
Tumbarello et al.^(59)^	2013	ICU, Italy	Retrospective cohort study	VAP diagnosed by BAL with organisms with COS	Colistin Jet or ultrasonic nebulizers; IV = 104 IV + IA-104	IV *Acinetobacter* 72/104 (69%) *Pseudomonas* 24/104 (23%) *Klebsiella* 8/104 (8%) IV plus IA *Acinetobacter* 56/104 (54%) *Pseudomonas* 28/104 (27%) *Klebsiella* 20/104 (19%) All organisms were COS	IV 52/84 (62%) IV+IA 42/82 (51%) *p* = 0.08	Not reported	IV 57/104 (55%) IV+IA 72/104 (69%) *p* = 0.03	Not reported
Palmer and Smaldone^(22)^	2014	MICU SICU United States	Randomized double-blind placebo controlled	VAT ≥2 mL sputum/4 hours and organism on Gram stain	Jet nebulizer Placebo plus IV *n* = 18 Inhaled antibiotic plus IV *n* = 24 Inhaled antibiotics included vancomycin and/or aminoglycoside determined by Gram stain IV antibiotics were all chosen by the responsible physician	Predominantly MDRO including MRSA and Gram-negative MDRO	IA+IV 26/27 (96%) isolates Placebo + IV 2/23 (9%) isolates	IA+IV 0/16 (0%) of new resistance to aerosolized drug, 2/16 (13%) new MDRO Placebo + IV 6/11 (56%) new resistance	IA + IV vs. Placebo + IV CPIS deceased significantly only in IA, *p-* = 0.0008	Creatinine similar in both groups at end of trial, no renal toxicity
Kollef et al.^(5)^	2016	ICUs in Europe, Middle East, and United States	Randomized double-blind placebo controlled	VAP with clinical diagnosis and Gram-negative organisms in BAL or mini BAL	Vibrating mesh plate- AFIS ^a^Placebo plus IV carbapenem vs. amikacin fosphomycin and IV carbapenem	Gram-negative organisms	IA and IV = 1/12, Placebo plus IV 8/29	Among patients without microbiological eradication the MICs showed a fourfold increase in AFIS IA plus IV vs. placebo 8 in (*p* = 0.02)	No difference in CPIS between groups	No increase in renal toxicity in the IA plus IV group vs. placebo and IV
Hassan et al.^(45)^	2018	CT ICU Cairo	Randomized Aerosol vs. IV amikacin as adjunctive therapy with systemic therapy	HAP and VAP	Pneumatic nebulizer for intubated patients Spontaneous breathing patients	MDR Gram-negative organisms 16/47	NA	NA	Inhaled 79/86 IV amikacin 33/47 *p* = 0.002	Decline in creatinine clearance Inhaled amikacin 12/86 Adjunctive IV amikacin 16/47 *p* = 0.001
Niederman^(11)^	2019	Multisite ICUs, 25 countries	Randomized double-blind placebo controlled	VAP with Gram-negative organisms	Synchronized vibrating mesh nebulizer; Placebo plus IV antibiotics, N-253; amikacin plus IV antibiotics, N-255	Gram-negative organisms	IA + IV: *P. aeruginosa* 65/75 (73%); *A. baumanii* 46/77 (60%); Placebo + IV: *P. aeruginosa* 43/88 (50%); *A. baumanii* 43/69 (62%)	Not described	IA + IV: 149 (58%) Placebo + IV: 145 (57%) *p* = 0.43	Device-related ventilator circuit occlusion 2/712 Cardiac arrest 2/712

Note: Only the two most common Gram-negative organisms are shown.

Modification of Table from Palmer LB: Aerosolized antibiotics for ventilator-associated infections. Chapter 10.4. In: Dhand R, editor. Textbook of Aerosol Medicine. Knoxville (TN): International Society of Aerosols in Medicine; 2015. p.e1–28. Available from: www.isam.org

^a^ A proprietary amikacin BAY41-6551 (NCT01004445).

AFIS, amikacin and fosphomycin inhalation system; COS, colistin only susceptible; CPIS, clinical pulmonary infection score; HAP, hospital-acquired pneumonia; IA, inhaled antibiotic; ICU, intensive care unit; IV, intravenous; MDR, multidrug resistant; MDRO, multidrug-resistant organisms; MRSA, methicillin-resistant Staphylococcus aureus, RCT, randomized controlled trial; VAP, ventilator associated pneumonia; VAT, ventilator associated tracheobronchitis.

For all trials given in Table 2, the dose placed in the nebulizer is described but pretrial data on the dose delivered to the lung are only present in a few.^(5,6,21,22,30)^ Furthermore, only a few trials examine the eradication of causal bacteria and emergence of resistance.^(5,21,22,42)^

To our knowledge, in most recent trials of inhaled antibiotic therapy to the intubated patient that have failed either in clinical response or eradication of causal bacteria, the delivery device was not characterized or often not even mentioned. Although “failure to assess the delivery device” stands out as an important omission in the general literature, this explanation seems inadequate when considering the two recent randomized Phase 2 (IASIS) and Phase 3 trials (INHALE II).^(5,6)^ In both, the devices were characterized in advance of the clinical trials in terms of deposition, particle size, and antibiotic concentrations delivered to the airway.^(30,60)^

## IASIS and INHALE Trials

### Design and outcome of IASIS and INHALE trials

The Phase 2 RCT (IASIS) conducted by Kollef et al. in 2016 administered a combination of amikacin and fosphomycin through a proprietary drug–device combination to ventilated patients with Gram-negative VAP.^(5)^ All patients received IV meropenem or imipenem for Gram-negative coverage for 7 days, and longer if clinically indicated. The endpoints are included in Table 3. No significant differences were found between active drug and placebo in any of the endpoints except in the culture data with a reduction in positive cultures.

**Table 3.** IASIS Endpoints for Active Drug (*n* = 71) and Placebo (*n* = 71)^(4)^

	Primary end point		*p*
CPIS baseline (mean ± SD)	5.6 ± 1.5	5.5 ± 1.6	NS^a^
CPIS day 10 (mean ± SD)	5.0 ± 3.1	4.8 ± 3.4	0.81

	Secondary hierarchal composite endpoint of mortality and time to clinical cure (no. of patients)		*p*
Mortality first	10	10	0.68
Clinical cure	15	18	

	Secondary hierarchal composite endpoint of mortality and ventilator-free days (no. of patients)		*p*
Mortality first	10	10	0.06
Ventilator-free days	13	27	

	Positive cultures		*p*
Tracheal cultures at day 1: Positive for Gram-negative organisms	19 (27)	40 (56)	0.001
Tracheal cultures at day 7: Positive for Gram-negative organisms	12 (17)	29 (41)	0.002

The reported tracheal aspirate concentrations for amikacin and fosfomycin, respectively, were 7720 μg/mL and 2430 μg/mL on day 3 and 7782 μg/mL and 2685 μg/mL, respectively, on day 10.

^a^ Actual *p* value not in publication.

The second major trial was INHALE II.^(6)^ This was a placebo controlled randomized trial of inhaled amikacin delivered as an adjunct to systemic therapy in mechanically ventilated patients with Gram-negative pneumonia. Both groups received appropriate systemic antibiotics as guided by the 2005 ATS Guidelines for VAP.^(61)^ The study failed to reach both primary (survival at days 28–32) and secondary outcome measures (Table 4).

**Table 4.** Summary of INHALE Endpoints

	Primary endpoint		*p* ^a^
Survival at days 28–32	191 (75%)	196 (77%)	0.43

	Secondary hierarchal composite endpoint		
Early clinical response^b^	149 (58%)	145 (57%)	

	Secondary endpoints		
Attributable mortality	43 (67%)	36 (63%)	
Days on ventilator, mean (SD)	20.6 ± 10.1	20.2 ± 10.2	

	Microbiological endpoints % (range)		
Eradication at test of cure (days 17–19)	71% (60–80)	68.5 (50–100)	
Emergence of new pathogens	21 (8.2%)	34 (13.5)	

Active drug *n* = 255 Placebo (*n* = 253).^(4)^

^a^ *p* values only calculated for survival.

^b^ Composite endpoint based on CPIS on 3, 5, and 10th day (vs. baseline), the presence of empyema or lung abscess at days 3, 5, 10 and all-cause mortality.

### Microbiological data from IASIS and INHALE trials

Examination of the bacterial eradication effects of the two trials suggests that there may have been problems with delivery of bactericidal concentrations. For example, despite high concentrations of antibiotic, Table 3 indicates that 17% of IASIS patients were still infected at day 7. The results from INHALE are given in Table 4. In that study, the four most common pathogens were *Acinetobacter baumanii*, Eschericia coli, Klebsiella pneumonia, and *P. aeruginosa*.

Eradication was higher in the active arm for all but *A. baumanii*. However, the eradication rate for these four pathogens was never >75%. This result also implies that, in the clinical arena the delivery device may not have reliably delivered adequate doses to all areas of infection, although the reported concentrations in the tracheal aspirates were also many times the MIC of the organisms.

This assessment is supported by observations from other clinical studies wherein the investigators rigidly controlled device and delivery conditions during their trials.^(21,22)^ Palmer and colleagues in two placebo controlled trials used a tightly controlled form of jet nebulizer delivery in patients with VAT or VAT with VAP that resulted in both clinical and bacteriological success, including complete eradication of pathogens in tracheal aspirates at end of treatment. Furthermore, in patients with follow-up cultures, from 1 to 4 weeks post-treatment, there was still no growth. They also found that inhaled therapy prevented the progression from VAT to VAP.

## Designing Clinical Trials for Ventilator-Associated Infection

### Current trial designs may be cause of failure

In VAP trials, the patient populations usually have well-established pneumonia. The severity of illness, prior exposure to antibiotics, and the length of time on ventilation may have contributed to the treatment failure in the IASIS and INHALE 2 trials.^(5,6)^ In the IASIS trial, the authors themselves indicated that failure to show a treatment difference between arms might have been secondary to late initiation of aerosol therapy. Many of their patients had received up to 6.6 days of intravenous therapy (for nonrespiratory sites) before initiation of inhaled therapy.

Supporting this theory, they also noted that the United States component of the study had more robust changes in CPIS and these patients had received only 3 days of IV antibiotics before inhaled therapy. However, this improvement was not associated with better clinical cure or secondary hierarchical mortality effect. Furthermore, in subgroup analysis of randomized patients in the ICU for <5 days and receiving <2 days of prior IV antibiotic, there was a much more robust change in CPIS in both arms (3 points lower in active arm and 2.5 points lower in placebo), suggesting that earlier treatment may have led to more robust results.

In the INHALE trial, analysis of the clinical data indicates that their patients also had advanced disease.^(6)^ In the intention to treat population, 167 of 354 of the active arm patients and 172 of 358 of placebo patients had Apache scores >20. Furthermore, pneumonia-attributable mortality was unusually high. For patients who received active drug, the pneumonia-attributed mortality was 67.2%, and in the placebo group, mortality was 63.2%. Most other studies report mortality rates of only 9%–13%.^(25,62,63)^ These differences suggest unusual severity of pneumonias in the INHALE population of patients. A mortality endpoint in the ICU is a high bar for any form of therapy.

The severity of illness in these ventilator-related infections, the antibiotic therapy given before enrollment in some cases, the failure of the inhaled treatment to eradicate organisms, and the insensitivity of mortality to different antibiotic therapies forecast the failure of these trials.

### IASIS and INHALE devices

Therapeutic trials of antibiotics require reasonable control of the delivered dose. This can be difficult in the intubated patient.^(64)^ Two major factors that might interfere with drug delivery are the device itself or the influence of the ventilator.

Both INHALE and IASIS used vibrating mesh nebulizers. Mesh devices are electronic and unlike jet nebulizers do not require added flow to the ventilator circuit. The devices used were proprietary, and detailed studies documenting drug delivery with repeated use are not available. The INHALE trial used Aerogen technology, the pulmonary drug delivery system (PDDS), which was breath actuated.^(6)^ In recent studies of the Aerogen Solo, a device similar to that used in the INHALE study but not breath actuated, a random failure rate of 30% was found in 40 experimental runs testing these devices on the bench.^(65)^

Additional studies during mechanical ventilation reported high residual nebulizer volumes or not nebulizing at all. Failure of gravitational feed was noted as well as bubble formation on the mesh particularly when used during mechanical ventilation.^(66,67)^ In the IASIS trial, an inline vibrating plate electronic nebulizer (eFlow Inline System; PARI GmbH) was used.^(5)^ Rottier et al. tested PARI eFlow devices and found >50% of the time the eflow switched off after 19 minutes.^(68)^

Ventilator effects are complex and will not be reviewed here in detail. The major factor affecting delivery is the duty cycle, the fraction of the breath taken up by inspiration. Breath actuation minimizes duty cycle effects. In INHALE, the PDDS was breath actuated, but in IASIS, the eflow was not. In IASIS, changes in ventilator settings may have affected drug delivery.

Although data from INHALE and IASIS are limited, tracheal antibiotic concentrations reported in INHALE were very variable ranging from 2890 to 41,602 mg/L. Such variability suggests inconsistent delivery.^(6)^ Better control of antibiotic levels has been reported with alternative breath-actuated delivery systems. Using a jet nebulizer, Miller et al. found that variability in antibiotic concentration in tracheal secretions could be tightly controlled with breath actuation and humidifier bypass.^(69)^

### Inhaled therapy: effects on bacterial resistance

An important metric in all future trials of inhaled antibiotics is the emergence of new resistance to the drug administered. Although systemic therapy is the only recommended treatment for ventilator-associated infection, there is a direct relationship with the amount of systemic antibiotics prescribed and the emergence of increased resistance. This fact, compounded with relatively poor cure rates, is the situation we are currently forced to accept.

If inhaled antibiotics could reduce the use of systemic antibiotics for respiratory infections, which are responsible for >50% of antibiotic use in the ICU, their use could reverse the increase in resistance seen today.

A common misconception is that aerosolized antibiotics increase bacterial resistance. Table 2 shows data from modern studies that were markedly different in design from the distant trials of the 70s that gave inhaled therapy a bad reputation.^(70)^ Between 2008 and 2017, five RCTs and one case–control study analyzed post-treatment cultures and found no increase in resistance in patients treated with aerosol therapy.^(5,21,22,42,46,48)^ Our group, using a well-characterized and robust aerosol delivery system, changed the spectrum of resistance in the intensive care unit.^(22)^ In our trials, inhaled antibiotics eradiated all pathogens including multiple drug-resistant organisms.^(21,22)^

All patients who acquired resistant organisms post-treatment received only systemic antibiotics. Similarly, Lu et al.'s randomized trial of intravenous versus inhaled antibiotics (as exclusive treatment) also showed the emergence of resistance only in the comparator group that received systemic antibiotics.^(46)^ Finally, in the IASIS trial, cultures that remained positive (12 of 71 active drug patients and 29 of 71 patients in the placebo group) were studied for the emergence of resistance.

The MICs of these cultures were compared with the MIC at the time of randomization.^(5)^ Of these cultures, 1 of 12 in the active drug group and 8 of 29 in the placebo group had a fourfold increase in MIC during the trial. The emergence of resistance to amikacin in the INHALE study in patients receiving inhaled active drug is of great interest, but those data have not been published.

### Future therapy

As we have already outlined, it is our opinion that studies to date may have failed because of either device technology or protocol design. The indications for treatment and the endpoints should be reconsidered in view of the data already summarized. Treating with adjunctive therapy for VAP (the approach used for both INHALE and IASIS) may be too late in the course of infection. Therefore, what is the optimal time to begin therapy?

Can we treat early tracheobronchitis and avoid well-developed VAP? Will inhaled therapy mitigate the need for systemic antibiotics and reduce resistance? Placebo-controlled trials designed to treat tracheobronchitis in patients identified as high risk for VAP, if successful, could answer these questions. The model in Figure 1 predicts localized inflammation and infection after a few days of intubation. What evidence supports earlier treatment?

Falagas et al., in a meta-analysis, reviewed the literature from 1950 to 2005.^(71)^ Of the 12 trials that could be considered prophylactic for VAP, there were 8 investigations that were either RCTs or prospective comparative trials. Aerosolized gentamicin was used in three trials, polymyxin in two trials, tobramycin in one trial, and ceftazidime in one trial. There were 1877 patients included in the meta-analysis. Primary outcomes included incidence of VAP and mortality.

An important secondary outcome was colonization with *P. aeruginosa*. Analysis of five RCTs demonstrated a reduction in VAP in the treated patients with an odds ratio (OR) of 0.49 (95% CI 0.32–0.76). Falagas and colleagues also included two nonrandomized trials, which yielded similar results for VAP. The latter studies were of added interest because there was a reduction in VAP in patients colonized with *P. aeruginosa* (OR, 0.51; 95% CI 0.30–0.86). A more recent systematic review and meta-analysis in 2018 by Pvoa et al. demonstrated that prophylactic antibiotics administered through the respiratory tract reduced the occurrence of VAP when compared with placebo or no treatment (OR 0.53; 95% CI 0.34–0.84).^(72)^

This effect was seen only when antibiotics were given by nebulization (OR 0.46; 95% CI 0.22–0.97), but not when they were administered by intratracheal instillation (OR 0.57; 95% CI 0.28–1.15). Although suggestive, none of these studies were designed to lead to formal approval of inhaled therapy, so universal availability of these protocols would be difficult to implement.

Putting all these analyses together, early treatment of tracheobronchitis targeted to the airways seems to have most potential for success. A modern early treatment trial would require a device designed to work with all commonly used ventilators with reproducible dosing in most settings. This is a high bar because ventilator circuits/humidifiers are not standardized and there is an interaction between aerosol delivery systems and the ventilator circuit that is difficult to control. However, the combination of consistent dosing in all patients and early treatment may be the best approach to preventing pneumonia with the added benefits of reducing the use of systemic antibiotics and bacterial resistance in critically ill patients. Potential benefits are listed in Table 5.

**Table 5.** Future Outcomes for Inhaled Antibiotics That Prevent Ventilator-Associated Pneumonia and Its Sequelae

Decreased need for initiation of systemic antibiotics for respiratory infection during the trial
Decreased emergence of resistance post-treatment both in the respiratory sites and nonrespiratory sites
Decreased daily dose of systemic antibiotics in the ICU
Increased ventilator free time Decreased antibiotic related diarrhea and specifically *Clostridium difficile* colitis

## Conclusion

We believe that the way forward is early intervention in airway infection. We have emphasized (1) early treatment is given before highly resistant organisms are present, as opposed to after they are present, (2) early treatment reduces the chance of bacterial resistance, (3) delivery is more effective at proximal sites of infection, (4) early topical therapy may avoid the use of systemic therapy, and (5) clinical trial design is facilitated because early treatment uses the development of pneumonia as an endpoint rather than mortality, which is likely unattainable in any ICU study of antibiotics.

## Notes

Reviewed by:

Stephan Ehrmann

James Fink
